# Assesment of Salivary and Serum Levels of HBD2 in Patients with Chronic Angioedema

**DOI:** 10.3390/jcm13247552

**Published:** 2024-12-11

**Authors:** Maja Štrajtenberger, Liborija Lugović-Mihić, Asja Stipić-Marković, Marinko Artuković, Ana Glavina, Nika Barbara Pravica, Milena Hanžek, Tamara Sušić, Andrea Tešija Kuna, Lara Nađ Bungić

**Affiliations:** 1 Department of Pulmology, Special Hospital for Pulmonary Diseases, 10000 Zagreb, Croatia; maja.strajtenberger@pulmologija.hr (M.Š.); laranadj.ng@gmail.com (L.N.B.); 2 Department of Dermatovenereology, University Hospital Center Sestre Milosrdnice, 10000 Zagreb, Croatia; 3 School of Dental Medicine, University of Zagreb, 10000 Zagreb, Croatia; 4 Department for Respiratory Infections, Dr. Fran Mihaljević University Hospital for Infections Diseases, 10000 Zagreb, Croatia; asjastipic90@gmail.com; 5 Faculty of Dental Medicine and Health Osijek, 31000 Osijek, Croatia; marinko.artukovic@gmail.com; 6 Department of Dental Medicine, University Hospital of Split, 21000 Split, Croatia; glavina2014@gmail.com; 7 Department of Oral Medicine, Study of Dental Medicine, School of Medicine, University of Split, 21000 Split, Croatia; 8 Department of Emergency Medicine, University Hospital Center Sestre Milosrdnice, 10000 Zagreb, Croatia; nbpravica@gmail.com; 9 Department of Clinical Chemistry, University Hospital Center Sestre Milosrdnice, 10000 Zagreb, Croatia; milenanjegovan13@gmail.com (M.H.); tamara.susic@kbcsm.hr (T.S.); andrea.kuna@gmail.com (A.T.K.)

**Keywords:** human β-defensin 2, angioedema, chronic urticaria, biomarkers, saliva, inflammatory factors, mast cells, immune response

## Abstract

**Background/Objectives**: Human β-defensin 2 (HBD2) is a protein that plays an important role in activating the immune system by modulating spinal pathways and the inflammatory response. According to previous research, HBD2 was proven to be important in chronic spontaneous urticaria (CSU) (their values were significantly elevated in CSU patients, with a significant correlation between HBD2 levels and the percentage of peripheral basophils, suggesting that elevated HBD2 levels may be a potential marker of basophil and mast cell activation), which led us to additional research on the HBD2 molecule in isolated chronic angioedema. The aim of this research is to examine HBD2 values in the saliva and serum of patients with isolated angioedema, as a potential biomarker of the disease. **Methods***:* This cross-sectional study involved a total of 102 participants, involving three groups: 33 patients with isolated chronic non-hereditary angioedema (AE) (defined as sudden onset of localized edema without chronic urticaria), 33 patients with angioedema associated with chronic urticaria (CU+AE), and 35 healthy participants (controls, CTRL). They provided a saliva sample to determine HBD2 levels using an ELISA (Enzyme-Linked Immunosorbent Assay). Subsequently, a peripheral blood sample (serum) was taken from the participants to determine HBD2 levels using the same ELISA. **Results***:* Salivary HBD2 levels were significantly higher in those with CU+AE than in the CTRL (*p* = 0.019). While salivary HBD2 values differed between those with angioedema and CTRL, the serum HBD2 values did not. Also, no correlation between the levels of HBD2 in saliva and serum was found. **Conclusions**: Since we found that salivary HBD2 values were significantly higher in those with CU+AE than in CTRL, this points to a possible role of the HBD2 molecule in pathogenesis of AE (namely, that it induces degranulation in mast cells and vascular permeability, and has antimicrobial properties) Therefore, more research is needed to determine how reliable salivary HBD2 measurement is, as well as its significance.

## 1. Introduction

Human β-defensin 2 (HBD2) is a protein that plays an important role in activating the immune system, acting by modulating spinal pathways and the inflammatory response, thereby providing protection to the organism against various pathogens. Structurally, HBD2 is a cysteine-rich cationic peptide of low molecular weight, consisting of 41 amino acids, also known as skin antimicrobial peptide 1 (SAP 1). It is mainly produced by epithelial cells, keratinocytes, and macrophages through contact with microorganisms (bacteria, viruses, and fungi) or through the action of various pro-inflammatory cytokines. Oral keratinocytes produce and secrete HBD2 primarily after stimulation by pro-inflammatory cytokines or as a result of exposure to bacterial endotoxins. Thus, from just two to four hours after contact with pro-inflammatory cytokines, the synthesis of this protein significantly increases. The primary function is antimicrobial; the peptide kills bacteria and fungi. In vitro, HBD2 promotes inflammation by recruiting CD4+ T cells and macrophages through interactions with C-C chemokine receptor 2 (CCR2) and C-C chemokine receptor 6b (CCR6) [1]. It has also been established that HBD2 induces degranulation of mast cells through interaction with the G-protein-coupled receptor MRGPRX2 [2]. Importantly, HBD2 acts as a pro-inflammatory pruritogen. In patients with skin diseases, it interacts with Toll-like receptor 4 via MRGPRX2 activation, which in turn induces histamine-independent itching [3,4]. Additionally, it has been observed that β-defensins increase vascular permeability in vivo [4].

In patients with skin diseases, HBD2 and HBD3 are primarily secreted by keratinocytes in the skin. Furthermore, serum HBD2 levels are significantly elevated in patients with skin diseases, including atopic dermatitis and psoriasis (Table 1) [5,6]. In one study, high levels of HBD2 were measured in the serum of patients with CSU compared to a healthy control group [7]. CSU is a common inflammatory skin disease characterized by pruritic wheals and/or angioedema that persist for more than 6 weeks and are triggered by some physical or environmental stimulus (cold, heat, exercise, pressure, sunlight, vibration, water, etc.) [8,9]. Additionally, urticaria and angioedema are comparable skin reactions, typified by a transient, localized enlargement of mucosal membranes, subcutaneous tissue, and the deep dermal layer. Although it can affect any portion of the body, the periocular area, lips, oral mucosa, vaginal areas, other mucosal regions, and other localizations are where it most frequently appears. In extreme situations, the lining of the intestines and upper respiratory tract may also be impacted. Angioedema can occur on its own, in conjunction with urticaria, or as a characteristic of anaphylaxis in disorders mediated by mast cells or bradykinin. It can also arise in conditions whose mechanisms are unknown, such as infections, uncommon diseases, or idiopathic angioedema. According to one study of HBD2 analysis using the Dunnett T3 test, CSU patients with associated angioedema had higher serum HBD2 levels than those without angioedema and the healthy control group [7]. Serum HBD2 levels did not differ between the healthy group and CSU patients without angioedema, however. In CSU patients, there was a negative correlation between the percentage of peripheral basophils and serum HBD2 levels. Moreover, no meaningful association between the severity of the disease and HBD2 levels was discovered (UAS7). Angioedema in CSU patients was significantly correlated with higher HBD2 levels (>72 pg/mL) and higher UAS (≤28), according to multiple logistic regression, while angioedema incidence was influenced by age, sex, and vitamin deficiency [7].

**Figure 1:**
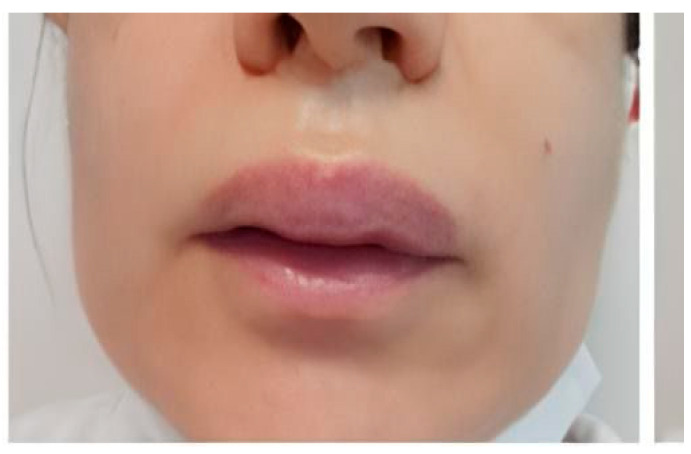
Isolated chronic angioedema.

In clinical work with chronic urticaria and non-hereditary angioedema (Figure 1), given the high risk of emergencies, it is crucial to examine the relevant disease biomarkers to guide early intervention and improve patient outcomes. The most recent recommendations for the diagnosis and treatment of CSU and non-hereditary angioedema state that the differential blood count, C-reactive protein, erythrocyte sedimentation rate, IgG anti-TPO, and total IgE levels should be checked in the management of these patients [5,6,7,8].

Mast cell activation is a key factor in the pathogenesis of CSU and chronic angioedema. This activation is well-known to involve regulators of FcεRI, IgE- independent pathways [e.g., G-protein-coupled receptor X2 (MRGPRX2)], tetraspanins, and the CD300 protein family [13]. In patients with CSU, thyroid proteins, nuclear antigens (double-stranded DNA), and IL24 promote mast cell activation through FcεRI cross-linking [14]. Viral and parasitic infections are significant triggers for CSU [8]. HBD molecules not only combat infections (exhibiting antimicrobial properties), but also act as mast cell secretagogues, eliciting clinical manifestations (neurogenic inflammation, pain, and itching) through a non-FcεRI cross-linking mechanism [2,4,15,16]. Specifically regarding CSU, Cao et al. discovered that serum levels of HBD2 were significantly elevated in CSU patients, with a significant correlation between HBD2 levels and the percentage of peripheral basophils, serum levels of translational tumor control protein (TCPA), and vitamin D levels compared to those in a healthy control group. TCPA is a well-studied active factor in histamine release in patients with allergic diseases, including asthma and CSU [17]. In the skin of CSU patients, an oxidative tissue environment rich in cytokines (caused by the activation of various inflammatory cells in CSU) and autoimmunity (reflected by the presence of IgG antibodies to FcεRα) can lead to a conformational change in TCPA to an active dimeric form [18]. Notably, levels of the dimerized form of TCPA are increased and cause the degranulation of mast cells and the activation of basophils (with or without IgE sensitization) [18]. Peripheral basopenia in CSU patients indicates the activation of skin basophils [19]. A correlation has been observed between HBD2 levels and reduced basophil counts and higher TCPA levels in CSU patients, suggesting that elevated HBD2 levels may be a potential marker of basophil and mast cell activation. Kanda and Watanabe proposed that there is no positive feedback loop between histamine and HBD2 levels in patients with inflammatory skin diseases [20]. Histamine synergistically increased the production of HBD2 in human keratinocytes. Since HBD2 can stimulate the release of histamine from mast cells and chemotaxis via TNF-α-activated neutrophils, a paracrine loop may occur between the levels of HBD2 and histamine, which could enhance the interaction between keratinocytes and mast cells or other inflammatory cells in the skin of patients with CSU [20,21].

Data suggest that skin inflammation caused by persistent infections (due to the activation of mast cells, basophils, macrophages, and/or T lymphocytes in the dermis of patients with CSU) can increase the production of HBD2 in CSU. According to the findings of Cao et al., higher UAS7 levels and HBD2 levels are associated with angioedema, although HBD2 levels are not significantly related to disease severity (UAS7) [7]. It has also been shown that HBD2 may be involved in the pathogenesis of angioedema in CSU patients, although HBD2 does not significantly enhance itching and urticarial formation. However, the expression of HBD2 in the skin was not measured, indicating that further research is needed to clarify how HBD2 levels increase in these patients, especially those with angioedema. Comparing HBD2 protein levels and gene expression between CSU patients and a healthy control group is necessary, as well as investigating specific pathways (histaminergic, cholinergic, or others) associated with elevated HBD2 levels in CSU patients.

HBD2 levels have been analyzed in serum and saliva. Insights regarding HBD2 levels in saliva are also important for some patients and disorders, such as chronic periodontitis and gingivitis. Thus, previous research has shown elevated levels of HBD2 in the saliva of patients with periodontal diseases compared to healthy individuals, making HBD2 a potential biomarker for the detection and prevention of periodontal diseases [22,23,24]. Additionally, HBD2 may serve as a marker of inflammation with potential therapeutic effects (inflammation suppression, reduction in oxidative stress, infection fighting) [25]. Diseases for which HBD2 is believed to have therapeutic potential include viral diseases, atopic dermatitis, allergic diseases, atopic asthma, oral lichen planus, wound healing, cell damage caused by smoking, and premature birth [25]. Its primary antimicrobial function is crucial, as is its involvement in the chemotaxis of immune cells and the activation of Toll-like receptors (TLRs) on their surface, along with its strong binding ability to complement component C1, which induces mast cell degranulation through interaction with MRGPRX2 and increases vascular permeability in vivo.

Given that a recent study showed higher serum levels of HBD2 in patients with CSU and associated angioedema compared to those with CSU without angioedema, this suggests a potential role for HBD2 in the pathophysiology of angioedema [7]. To date, no studies have examined the correlation between HBD2 levels in saliva and serum in patients with angioedema in relation to the severity of the clinical presentation, nor have they compared HBD2 levels in saliva and serum with coagulation factor levels. Other conditions where elevated salivary HBD2 levels have been observed include periodontal diseases, H. pylori infections, inflammatory bowel diseases, atopic dermatitis, psoriasis, and lichen sclerosis.

Overall, taking into account the numerous properties of this molecule, the current evidence suggests that HBD2 is a potential marker of inflammation and also a molecule with a potential therapeutic effect (suppression of inflammation, antimicrobial activity, reduction in oxidative stress, etc.), which points to its potential significance for recurrent angioedema and CSU.

The aim of this research is to examine HBD2 values in the saliva and serum of patients with isolated angioedema, as well as to obtain insight into HBD2 as a potential biomarker of the disease.

## 2. Materials and Methods

### 2.1. Participants

This cross-sectional study involved a total of 102 participants in three groups: 33 patients with isolated chronic non-hereditary angioedema (defined according to the guidelines of Zuberbier et al. from 2022 [8]), who met the inclusion and exclusion criteria; 33 patients with non-hereditary angioedema associated with chronic urticaria; and 35 healthy participants who met the exclusion criteria. We did not prospectively enroll the patients. The period of patient enrollment in the research lasted from December 2022 to June 2024.

Criteria for the inclusion of the test group is as follows: Adult patients with diagnosed angioedema based on valid guidelines for the diagnosis of angioedema (according to Zuberbier et al., 2022 [8]) who signed an informed consent form. Thus, statistically, if the mean differences in serum HBD2 between healthy and chronic urticaria subjects are expected to be 21.8 with a standard deviation of 28.6 in urticaria and 27.4 in healthy subjects, a minimum sample of 27 participants per group is needed to detect significant differences, taking into account a power of 80% and a significance level of 0.05 [7].

Exclusion criteria of the test group: 1. isolated CSU without angioedema; 2. application of systemic antimicrobial drugs, antineoplastic therapy, corticosteroids and immunosuppressive agents (15 days before inclusion in the study); 3. smoking (due to effect of nicotine on the level of salivary HBD2); 4. diseases of the oral mucosa, gastroesophageal reflux, or Sjögren’s syndrome; 5. autoimmune diseases, malignant diseases, or diabetes; 6. diseases of the upper respiratory tract or other infectious diseases (30 days before inclusion in the study); 7. patients on anticoagulant therapy; 8. patients with prostheses and orthodontic appliances; 9. active periodontal disease; and 10. pregnancy and breastfeeding.

All patients were examined by an allergist/immunologist at the Outpatient Clinic for Allergology and Clinical Immunology at the Rockefeller Special Hospital for Lung Diseases. After an allergist’s examination, they received a recommendation for treatment and were introduced to the research being conducted at the Rockefeller Special Hospital for Lung Diseases and were offered to participate in it. Along with verbal instructions about the methods and purpose of the research, they were reminded that participation was voluntary, anonymous, and that they could withdraw at any time. After verbal instructions, subjects were asked to read the voluntary consent and, if they agreed, sign it.

Also, all subjects were referred to a doctor of dental medicine for an examination to rule out oral disease (active periodontal disease), and those with a positive finding were included in the research.

Patients who agreed to participate and met the inclusion criteria were included in this study.

They provided a saliva sample together with the peripheral blood sample to determine HBD2 concentrations using the ELISA (Enzyme-Linked Immunosorbent Assay). Saliva was collected in two tubes within five minutes for each participant.

Subsequently, a peripheral blood sample (serum) was taken from the participants to determine HBD2 levels using the ELISA, following the manufacturer’s instructions, and the samples were stored at −70 °C.

The research was approved by the Ethics Committee of University Hospital Center “Sestre Milosrdnice”, Zagreb, Croatia, in December 2022; number of protocol: 251-29-11-21-08, for studies involving humans.

### 2.2. Saliva Sampling and Analysis for HBD2

At the initial examination, all participants were instructed to refrain from intense physical activity and psychological stress for three days prior to the saliva sampling. They were also asked to avoid eating, drinking, and brushing their teeth for 90 min before the sampling procedure. Total unstimulated saliva was collected from participants in the morning between 9 and 10 a.m. to minimize daily variations. A systematic review and meta-analysis [26] indicated that previous studies collecting both types of saliva (unstimulated and stimulated) showed similar results in levels of salivary biomarkers [26]. This study confirmed that unstimulated saliva is sufficient for detecting salivary biomarkers and may be relevant in future research [26].

During saliva sample collection, participants were instructed to comfortably position themselves with their heads slightly tilted forward. Immediately before saliva sampling, all participants rinsed their mouths with water to avoid contamination from other sources, then waited ten minutes before collecting the sample. They were instructed to swallow their saliva just before the collection began. Approximately 2.50 mL of saliva was collected from participants (angioedema group, angioedema with urticaria group, and control participants) in graduated tubes (Salivette) (1534.500, SARSTEDT AG & Co. KG, Nümbrecht, Germany) using the “spitting method”. In this method, the participant held saliva in their mouth for 60 s and then spat it into the graduated tube. This process was repeated for another ten minutes. Participants did not use any materials to stimulate saliva secretion.

### 2.3. Serum Sampling and Analysis for HBD2

After collecting the total unstimulated saliva sample for the analysis of salivary HBD2 concentration, blood samples were taken from the participants to determine serum HBD2 concentration using the same ELISA.

Saliva samples were delivered to the Department of Medical Laboratory Diagnostics at the “Dr. Fran Mihaljević” Clinic for Infectious Diseases, where the Salivette tubes were stored at −70 °C until analysis. To determine salivary HBD2 concentration, the frozen samples were first thawed at room temperature for 30 min. They were then centrifuged at 1500× *g* for five minutes, and the supernatant was used for analysis. Blood samples were taken from participants in tubes without anticoagulant. After 30 min, these samples were centrifuged at 2500× *g* for 10 min. Following centrifugation, the serum samples were aliquoted and frozen at −70 °C until analysis. The concentration of salivary and serum HBD2 was measured using the ELISA with a commercial reagent kit from MyBioSource (Inc., San Diego, CA, USA). The manufacturer specifies a test sensitivity of 3.9 pg/mL and linearity from 7.8 to 500 pg/mL (samples with concentrations > 500 pg/mL were diluted and repeated in a new series). The declared coefficients of variation (CV, %) within a series were 4.5, 4.6, and 3.9 for concentrations of 93, 210, and 460 pg/mL, and between series were 5.3, 5.9, and 6.4 for concentrations of 89, 206, and 455 pg/mL. The method is based on a sandwich immunoassay principle. Antibodies specific to HBD2 are bound to the wells of a microtiter plate. Upon adding serum or saliva, HBD2 from the sample binds to the specific antibodies in the wells. After washing away the unbound fractions, a detection antibody specific to a different HBD2 epitope, conjugated with biotin, is added. After incubation, streptavidin conjugated to an enzyme (horseradish peroxidase, HRP) is added, which binds to biotin. After washing away unbound fractions, a substrate (3,3′,5,5′-Tetramethylbenzidine, TMB) is added. The intensity of the developed color is proportional to the concentration of HBD2. The ELISA was performed using the VirClia ELISA/CLIA analyzer (Vircell, Granada, Spain).

The presence of blood in saliva (hemolysis) was tested by visual reading, and saliva samples containing blood were excluded from the study [27]. Hemolytic serum samples were also excluded from the study.

## 3. Results

**Figure 2:**
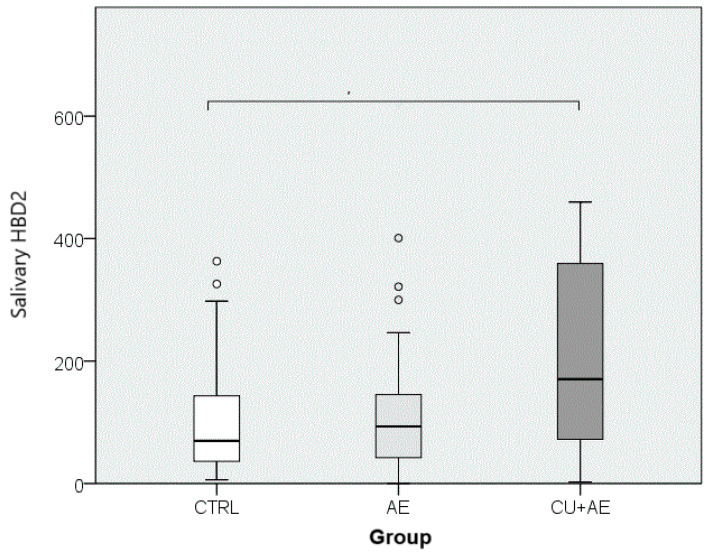
Comparison of salivary HBD2 between groups. (Circles indicate outlier values. Groups connected by a horizontal line are statistically significantly different). HBD2—human beta defensin 2; CTRL—healthy controls; AE—angioedema; CU+AE—angioedema associated with urticaria.

The results of the salivary HBD2 measurement in patients with angioedema compared to the control group (i.e., across three groups) are presented in Figure 2. The analysis of the obtained HBD2 values indicates that the groups of participants statistically significantly differed in terms of salivary HBD2 levels, with a small effect size (*p* = 0.019; ε^2^ = 0.079). When comparing the groups pairwise, salivary HBD2 levels were significantly higher in those with angioedema accompanied by urticaria (CU+AE) than in the control group (CTRL) (*p* = 0.019).

**Figure 3:**
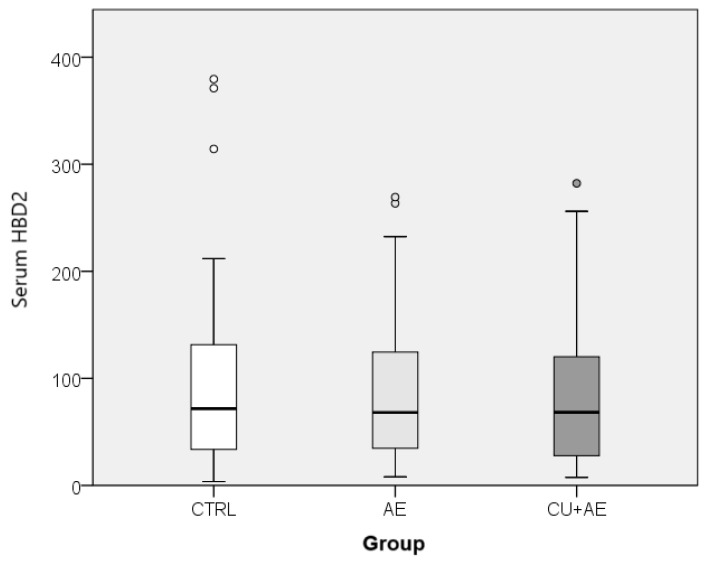
Comparison of serum HBD2 between groups. HBD2—human beta defensin 2; CTRL—healthy controls; AE—angioedema; CU+AE—angioedema associated with urticaria.

While the salivary HBD2 values differed among groups of patients with angioedema and healthy participants, the serum HBD2 values did not differ between them (Figure 3).

**Figure 4:**
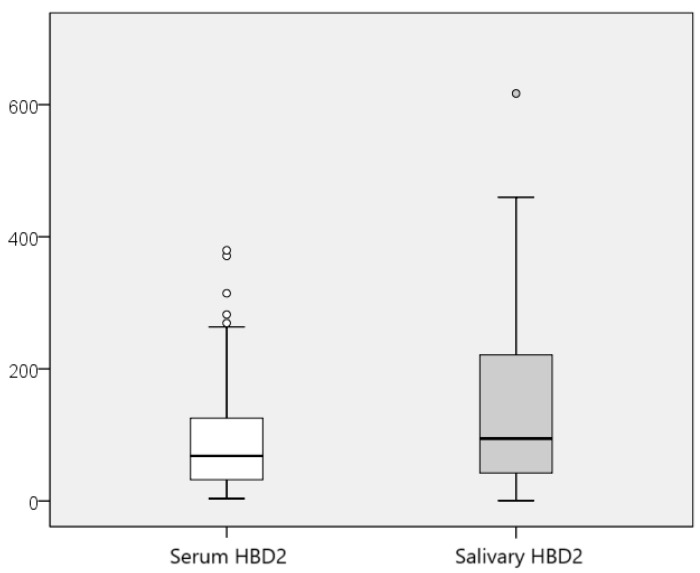
Comparative display of the range of values for serum and salivary HBD2.

The HBD2 values in saliva were also compared with those in serum of the participants. A comparative overview of the range of serum and salivary HBD2 values is presented in Figure 4.

**Figure 5:**
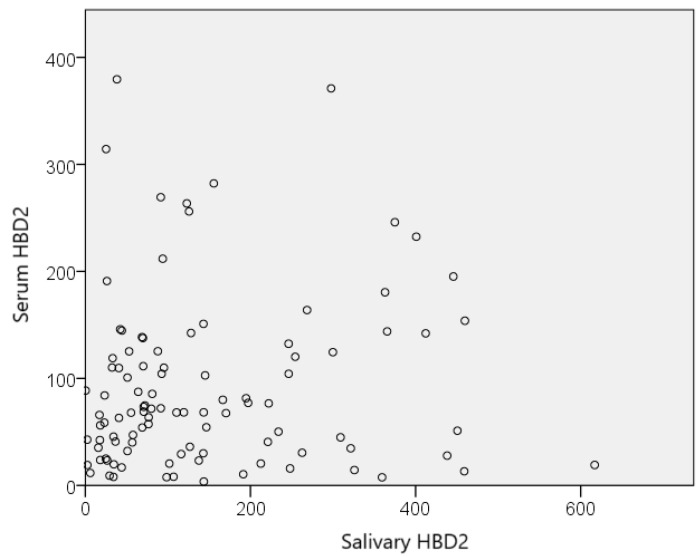
Relationship between serum and salivary HBD2 values. HBD2—human beta defensin 2.

Additional statistical analysis (scatter plot analysis and Spearman correlation) showed no correlation between the levels of HBD2 in saliva and serum (they did not correlate linearly), indicating that salivary HBD2 is not a good indicator of serum HBD2 values (Figure 5). Additionally, no correlation was observed between serum and salivary HBD2 values and other serum biomarkers.

## 4. Discussion

Based on the numerous roles of HBD2 and the recently determined higher values of HBD2 in patients with CSU, in this study, we aimed to investigate its levels in patients with chronic angioedema. As is known, β-defensins are antimicrobial peptides that contribute to the resistance of epithelial (mucosal/skin) surfaces to microbial colonization [28]. They have been confirmed in all mammalian species and are predominantly produced by epithelial cells lining various organs such as the skin epidermis, bronchial mucosa, oral mucosa, and the genitourinary tract. In humans, β-defensins like HBD2 induce the activation and degranulation of mast cells, leading to the release of histamine, prostaglandin D2, and other mediators [29]. It is also essential to mention genetic factors, as β-defensins are encoded by genes that produce antimicrobial peptides found in white blood cells such as macrophages, granulocytes, and NK cells [30,31]. Defensins can enhance the innate immune system and strengthen the adaptive immune system through the chemotaxis of immune cells (T-cells, monocytes, dendritic cells, and mast cells) to the site of infection [32].

HBD2 is a cationic peptide of low molecular weight, rich in cysteine (composed of 41 amino acids), produced by epithelial cells, keratinocytes, and macrophages, primarily upon contact with microorganisms (bacteria, viruses, and fungi) or the action of various pro-inflammatory cytokines [33,34,35]. It is also crucial for numerous immune functions of the body. Regarding its role, HBD2 is essential in activating the immune system, modulating signaling pathways, and the inflammatory response, as well as protecting the body from various microorganisms (primarily bacteria and fungi). In addition to its primary antimicrobial function, HBD2 may also be involved in the chemotaxis of immune cells and the activation of Toll-like receptors (TLR) on their surface. It also binds strongly to the C1 component of the complement system. Moreover, HBD2 promotes inflammation (in vitro) through recruiting CD4+ T cells and macrophages the complement system via interplay with chemokine receptors CCR2 and CCR6 [4,6]. HBD2 additionally induces mast regulated degranulation interplay with the G-protein coupled mast molecular receptor X2 (Mast-associated G-protein coupled receptor member X2, MRGPRX2) and will increase vascular permeability (in vivo). Thus, interactions between HBD2 and the mast cell receptor MRGPRX2 induce mast cell degranulation [2]. MRGPRX2 is a protein predominantly found on skin mast cells, sensory neurons, and keratinocytes, and its activation on mast cells leads to IgE-independent manifestations resembling type 1 hypersensitivity (pseudo-allergic reactions) [36].

The role of HBD2 has been demonstrated in several dermatological mucosal and allergic conditions. Regarding the production of defensins in the skin, human defensins HBD2 and HBD3 are primarily produced in keratinocytes, but have also been detected in serum. In patients with skin diseases, significantly higher serum levels of HBD2 have been observed, including atopic dermatitis and psoriasis [5,6]. It has been noted that in patients with skin diseases, HBD2 acts as a pro-inflammatory pruritogen, with the interaction between HBD2 and Toll-like receptor 4 promoting the activation of MRGPRX2, which induces itching independent of histamine [3,4]. In addition to these diseases, HBD2 is also essential in CSU, as skin inflammations with urticaria may be triggered or exacerbated by persistent infections (due to the activation of mast cells, basophils, macrophages, and/or T cells), which can increase HBD2 production in the dermis. It is also essential to highlight that CSU is often accompanied by angioedema, which may be linked to HBD2, as higher serum levels of HBD2 have been demonstrated in patients with CSU and accompanying angioedema compared to those with CSU without angioedema [7].

Since infections can be possible causes and/or triggers for CSU, the role of HBD molecules is also possible in this process. In addition to their antimicrobial activities, HBD molecules act as mast cell secretagogenes, inducing neurogenic inflammation, pain, and itching (through a non-FcεRI cross-linking mechanism) [2,4,8,14]. In patients with CSU, research has revealed significantly elevated serum levels of HBD2, which correlate with the percentage of peripheral basophils, and with the level of a serum protein called translationally controlled tumor protein (TCTP) and vitamin D, compared to a healthy control group [7].

A recent investigation conducted on patients with CSU found that serum levels of HBD2 were significantly elevated when compared to those of healthy individuals [7]. Furthermore, when assessing HBD2 levels in CSU patients experiencing angioedema (utilizing Dunnett’s T3 test), results indicate higher levels than in both the healthy group and CSU patients without angioedema; however, no difference in HBD2 levels was noted between CSU patients without angioedema and healthy individuals. In CSU patients, a negative correlation was identified between serum HBD2 levels and the percentage of peripheral basophils, yet no significant correlation was observed regarding HBD2 levels and the severity of the disease (UAS7). Nonetheless, the statistical analysis through multiple logistic regression revealed that elevated HBD2 levels (>72 pg/mL) and increased disease severity (UAS ≥ 28) were significantly linked to the occurrence of angioedema in CSU patients, while no associations were detected between angioedema and factors such as age, gender, or vitamin deficiency [7].

Since basophil counts are proven biomarkers of CSU severity (peripheral basopenia in these patients confirms skin basophil activation), it is essential to note the observed correlation between HBD2 levels and lower basophil counts, as well as higher TCTP levels. Therefore, increased HBD2 levels may be a potential biomarker of basophil and mast cell activation [19,37]. However, diagnostic biomarkers for CSU are still missing. Most biomarkers described so far do not appear to be specific enough for this disease. Basophenia and activation of the coagulation cascade could be biomarkers of disease activity and severity, but the information available so far is insufficient to consider their routine use.

When accompanying angioedema occurs with CSU, it is essential to address it, as it may be associated with HBD2. Most angioedemas in CSU patients are histaminergic, mediated by mast cells, and associated with itching [38]. Clinically, patients with CSU and angioedema exhibit more significant disease activity and longer disease duration compared to those without angioedema [39,40]. However, since CSU severity (measured using the UAS7 questionnaire) only assesses the presence of urticaria and itching without considering the presence of angioedema, it is essential to evaluate angioedema and its activity when assessing the condition and quality of life in CSU patients. Cao and colleagues found that greater CSU severity (higher UAS7) and HBD2 levels were associated with angioedema, while there was no significant correlation between HBD2 levels and CSU severity (UAS7) [7]. Thus, in CSU patients, HBD2 may be potentially involved in the pathogenesis of their associated angioedema (while HBD2 was not strongly linked to itching and urticaria formation). It is also reported that in patients with inflammatory skin diseases, there are no positive feedback loops between histamine and HBD2 levels [20]. According to research results, histamine synergistically increases HBD2 production in human keratinocytes when combined with TNF-α or IFN-γ expression. Given that HBD2 has the capability to provoke mast cells into releasing histamine and drawing in TNF-α-activated neutrophils, it is conceivable that within the skin of patients with CSU, a paracrine loop involving HBD2 and histamine concentrations may amplify the interactions between keratinocytes and mast cells or various other inflammatory cells [20,21]. Additionally, elevated levels of HBD2 have been noted in other conditions, suggesting possible therapeutic implications for diseases such as periodontal diseases, H. pylori infections, inflammatory bowel diseases, atopic dermatitis, psoriasis, and lichen sclerosis. Among the conditions where HBD2 may have a therapeutic effect are viral diseases, allergic conditions such as allergic asthma and atopic dermatitis, oral lichen planus, wound healing, cell damage from smoking, and preterm birth [25].

Additionally, oral production of HBD2 has been established, with epithelial cells of the gingival mucosa secreting it (confirmed by mRNA expression for HBD2 in these cells and its presence in saliva) [41,42]. Oral epithelial cells primarily produce and secrete HBD2 in response to pro-inflammatory cytokine stimulation or bacterial endotoxin activity after contact with pro-inflammatory cytokines (which happens rapidly, from just two to four hours), significantly increasing the synthesis of this protein [43]. Moreover, studies have shown that the level of HBD2 in the saliva of patients with periodontal disease is significantly higher than that of healthy people. This suggests that HBD2 can be used as a potential biomarker for detecting and preventing periodontal disease [22,23,24]. Due to insufficient data on HBD2 expression in the skin of patients with allergic mucocutaneous diseases such as CSU and chronic urticaria, further research is required to clarify the causes/etiology of increased HBD2 levels in these patients. This is particularly important for CSU, especially in patients with angioedema. Future research should include a comparison of HBD2 levels and the expression of related genes between CSU patients and healthy control groups, as well as an investigation into the potential connection between the pathogenic pathways of angioedema/urticaria (whether histaminergic, cholinergic, or otherwise) and elevated HBD2 levels in CSU patients. Considering this molecule’s numerous properties, current evidence suggests that HBD2 is a potential marker of inflammation and may also have therapeutic potential (anti-inflammatory, antimicrobial effects, reduction in oxidative stress, and more). This indicates its potential significance for recurrent angioedema and urticaria [25]. Our results for salivary HBD2 determination in patients with angioedema compared to the control group (i.e., across the three groups) and analysis of the obtained HBD2 levels show that the participant groups differed significantly in salivary HBD2 levels, although with a small effect size (*p* = 0.019). Comparing the groups in pairs revealed that salivary HBD2 was significantly higher in those with angioedema and urticaria compared to the control group (*p* = 0.019). Furthermore, salivary HBD2 values were positively correlated with age (*p* = 0.021), indicating an association between HBD2 levels and age.

Several studies have explored the value of HBD2 in allergic skin diseases, although only one has investigated serum HBD2 levels in patients with CSU associated with angioedema. However, no studies have examined serum or salivary HBD2 in patients with isolated angioedema [7,10,11,44,45,46,47,48]. This indicates that our results are the first obtained from investigating HBD2 levels in patients with isolated angioedema. Cao et al., in their 2021 study, demonstrated significantly elevated serum HBD2 levels in a group of CSU patients with associated angioedema compared to a healthy group and a group without angioedema [7]. Our results do not align with their findings, as they did not observe higher values. While our study found differences in salivary HBD2 levels between patients with angioedema and healthy participants, serum HBD2 levels did not differ. Importantly, we also compared salivary HBD2 levels with serum levels in the participants. Additional statistical analyses found no correlation between HBD2 levels in saliva and serum (no linear correlation), suggesting that salivary HBD2 may not be a good indicator of serum HBD2 levels in our participants. Furthermore, no correlation was observed between serum or salivary HBD2 levels and other serum biomarkers. Therefore, more research is needed to determine how reliable salivary HBD2 measurement is, as well as its significance. So, the strength of this research is that our results present the first data on HBD2 levels in patients with isolated angioedema, as well as being the first study which determined the value of HBD2 in the serum and saliva of patients with isolated angioedema. However, this study has potential limitations. When interpreting the results, we should take into account that the results may have been influenced by the fact that the sample size is limited and that we did not analyze and compare serum/salivary HBD2 levels with other serum biomarkers, due to lack of time and finances, as well as that we did not compare them with the severity of clinical indicators of the disease. Therefore, in future research, it would be desirable to compare serum and salivary values of HBD2 with other serum indicators and indicators of the clinical severity of angioedema.

Concerning the use of saliva for the research purpose, it could be mentioned that saliva contains a variety of proteins, immunoglobulins, enzymes, and hormones, making it an fantastic medium for biomarker discovery. Unlike blood, saliva collection is non-invasive and may be repeated, which is specially positive for both clinical practice and research. Salivary biomarkers reflect the immune and endocrine responses involved in the pathophysiology of many skin and mucosal diseases. Salivary biomarkers are a promising alternative to standard blood diagnostics because of their non-invasive detection capabilities and their ability to reflect both systemic and local immune responses. Important biomarkers along with cytokines, immunoglobulins, and stress-related hormones have been found in saliva and have shown correlations with diseases such as atopic dermatitis, psoriasis, systemic lupus erythematosus, oral lichen planus, and CSU. Despite significant progress, it remains challenging to standardize these biomarkers across diverse populations and integrate cost-effective diagnostic tools into clinical practice. Future studies are needed to validate these markers, develop reliable point-of-care devices, and create comprehensive biomarker panels to enable early intervention and improve patient outcomes. The ongoing developments in the fields of salivary proteomics and exosome profiling promise a transformative future for the noninvasive, precise diagnosis of skin diseases.

Since HBD2 exhibits antimicrobial and immunomodulatory activities, current CSU treatments include the normalization of immune status, as well as permeability and antimicrobial barrier function. Despite recent evidence suggesting a role for HBD2 in CSU pathogenesis, many efforts have been made to develop these peptides for therapeutic applications. Therefore, further studies are needed to reveal the role of HBD2 in CSU pathogenesis and evaluate its clinical potential, which will clearly benefit from new therapeutic approaches.

## 5. Conclusions

Since salivary HBD2 values were statistically significantly different between the groups and are much higher in those with chronic angioedema with urticaria than in controls, it indicates that HBD2 may be involved in the pathogenesis of chronic angioedema. Salivary HBD2 is not a good indicator of serum HBD2 values. Therefore, more research is needed to determine how reliable salivary HBD2 measurement is, as well as its significance.

## Figures and Tables

**Table 1:** Previous data on HBD2 obtained by studies on patients with dermatoses.

Authors	Participants	Examined Factors	Obtained Study Results
Ong et al., 2002 [10]	8 patients with moderate-to severe AD, 11 patients with psoriasis, and 6 healthy persons.	serum β-defensins	Significantly lower β-defensin levels in inflamed AD skin lesions.
Kanda et al., 2012 [5]	26 patients with AD and 27 healthy persons.	serum HBD2 samples	Higher serum HBD2 levels in AD patients than in healthy persons
Clausen et al., 2013 [11]	25 AD patients and 11 controls.	serum HBD2 and other factors	a significant correlation between HBD2, disturbed skin barrier function, and AD severity.
Li et al., 2017 [12]	18 AD-like GVHD patients, 12 LP-like GVHD patients, and 14 healthy persons.	skin HBD2 mRNA	Increased HBD2 mRNA expression in skin lesions of AD-like GVHD and LP-like GVHD patients.
Jin et el., 2017 [6]	18 psoriasis patients randomized to receive placebo or tofacitinib.	serum HBD2 levels	Significantly decreased serum HBD2 levels in patients treated with tofacitinib compared with baseline and placebo-treated patients.
Cao et al., 2021 [7]	124 CSU patients and 56 healthy persons.	serum HBD2 levels	Higher serum HBD2 levels in CSU patients than in healthy persons; higher values in those with angioedema than without angioedema.

Abbreviations: AD—atopic dermatitis; CSU—chronic spontaneous urticaria; GVHD—Graft-Versus-Host Disease; LP—Lichen Planus; HBD2—human beta defensin 2.

## Data Availability

The original contributions presented in this study are included in the article. Further inquiries can be directed to the corresponding author.
